# Newly Developed Semi-Solid Formulations Containing *Mellilotus officinalis* Extract: Characterization, Assessment of Stability, Safety, and Anti-Inflammatory Activity

**DOI:** 10.3390/pharmaceutics16081003

**Published:** 2024-07-29

**Authors:** Jovana Bradic, Anica Petrovic, Milos Nikolic, Nikola Nedeljkovic, Marijana Andjic, Nebojsa Kladar, Stefani Bolevich, Vladimir Jakovljevic, Aleksandar Kocovic

**Affiliations:** 1 Department of Pharmacy, Faculty of Medical Sciences, University of Kragujevac, 34000 Kragujevac, Serbia; jovanabradickg@gmail.com (J.B.); petkovicanica0@gmail.com (A.P.); milos.nikolic@fmn.kg.ac.rs (M.N.); nikola.nedeljkovic@fmn.kg.ac.rs (N.N.); salekkg91@gmail.com (A.K.); 2 Center of Excellence for Redox Balance Research in Cardiovascular and Metabolic Disorders, 34000 Kragujevac, Serbia; drvladakgbg@yahoo.com; 3 Department of Pharmacy, Faculty of Medicine, University of Novi Sad, 21000 Novi Sad, Serbia; nebojsa.kladar@mf.uns.ac.rs; 4 Center for Medical and Pharmaceutical Investigations and Quality Control, University of Novi Sad, 21000 Novi Sad, Serbia; 5 Department of Pathological Physiology, 1st Moscow State Medical, University I.M. Sechenov, 119991 Moscow, Russia; alistra555@mail.ru; 6 Department of Physiology, Faculty of Medical Sciences, University of Kragujevac, 34000 Kragujevac, Serbia; 7 Department of Human Pathology, 1st Moscow State Medical, University I.M. Sechenov, 119991 Moscow, Russia

**Keywords:** *Melilotus officinalis*, natural products, in vivo anti-inflammatory activity, molecular docking

## Abstract

*Melilotus officinalis* has been traditionally used as an anti-inflammatory agent; nevertheless, a comprehensive evaluation of its efficacy and safety and comparison with standard drugs are lacking. Taking into consideration concerns with current therapies, like efficacy limitations, side effects, and resistance, we aimed to develop a natural gel and cream based on *Melilotus officinalis* extract and explore their anti-inflammatory potential. After the chemical analysis of the extract confirmed the presence of coumarin, *p*-coumaric acid, gallic acid, and quercetin, formulations were prepared and subjected to physical and chemical stability evaluations over 6 months. The safety potential was tested in rats, while the anti-inflammatory activity was assessed both via in silico tests and in a rat model of inflammation. The examined formulations showed stable physical characteristics at the defined storage conditions and did not exert any sign of adverse skin reaction. The gel formulation exhibited a remarkable effect in inflammation reduction comparable with hydrocortisone. The in silico results suggest that coumarin, *p*-coumaric, and gallic acid bind to COX-1 and COX-2 with a lower affinity compared to diclofenac. On the other hand, quercetin demonstrated comparable inhibitory activity and stronger interaction compared to the control drug. Our results indicate that the examined formulations are stable and safe and may be promising dermal products for the alleviation of inflammatory skin conditions.

## 1. Introduction

In previous years, the pharmaceutical and cosmetic industries have turned to the development of formulations for skin care and treatment that are of a natural origin, thus highlighting the significance of using plant extracts [1]. Plant species with a long history of traditional use, in addition to a confirmed valuable phytochemical profile, represent promising active ingredients for dermocosmetic products [2]. *Melilotus officinalis* (*M. officinalis*, yellow sweet clover) is a widely distributed annual herb belonging to the family *Fabaceae*, genus *Melilotus*. For centuries, *M. officinalis* has been used in traditional medicine for the treatment of neuralgias and venous circulatory disturbances and as diuretic, sedative, and carminative [3]. On the other hand, when externally applied, this plant species exerts an emollient, phlebotonic effect and contributes to the relief of joint pain. Moreover, the benefits of *M. officinalis* topical usage involve the alleviation of skin inflammation and the treatment of bruises and sprains. The prominent healing properties of *M. officinalis* have led to its inclusion in the European Medicines Agency (EMA) catalogue, the British Herbal Compendium, and Hagers Handbuch der Pharmazeutishen Praxis [4].

Chemical analyses suggested the presence of various compounds in *M. officinalis*, such as coumarins, flavonoids, phenolic acids, vitamin C, tannins, and allantoin that contribute to the overall therapeutic potential of this plant. The coumarin content (up to 0.9%), such as dicoumarol, dihydrocoumarin, and umbelliferone, as well as flavonoids including kaempferol, quercetin, and rutin, are believed to contribute to the anti-inflammatory and soothing properties of *M. officinalis* [5,6,7].

Skin inflammation represents a body’s defense response to certain external stimuli, such as pathogen microorganisms, harmful substances, irritants, etc. Numerous researchers have attempted to create natural products for the treatment of skin inflammation in order to overcome the adverse and serious side effects related to currently available medications, such as corticosteroids and nonsteroidal anti-inflammatory drugs (NSAIDs) [8]. Previous studies predominantly suggest the in vitro anti-inflammatory properties of *M. officinalis* extract, while scientific data referring to the effects of the local application of *M. officinalis* extract alone or incorporated in certain vehicles are lacking.

Therefore, we aimed to develop and characterize semi-solid formulations based on *M. officinalis* extract and assess their stability, safety, and anti-inflammatory effects. The special focus of this research was based on the estimation of anti-inflammatory capacity through the integration of in silico and in vivo methods.

## 2. Materials and Methods

### 2.1. Plant Materials and Extract Preparation

Aerial parts of *M. officinalis* were obtained from The Institute for the Study of Medicinal Herbs “Dr Josif Pančić”, Belgrade, Serbia. The powdered aerial part of *M. officinalis* was extracted by maceration with 70% aqueous ethanol as a solvent. Ethanol was evaporated under reduced pressure (RV05 basic, IKA, Staufen im Breisgau, Germany), and the dry extract was used for the preparation of semi-solid formulations. 

### 2.2. Chemical Characterization of M. officinalis Extract 

The technique of high-performance liquid chromatography (HPLC) was used for the phytochemical characterization of *M. officinalis* extract. An Agilent Technologies 1100 device paired with a diode array detector (Agilent Technologies, Santa Clara, CA, USA) was used. Due to the specificity of the starting plant material, two different chromatographic procedures were used. A previously described procedure [9] was used for general analysis with special emphasis on the separation and identification of phenolic and flavonoid compounds. Briefly, a reversed-phase column (Nucleosil C18, size 250 mm × 4.6 mm and particle size of 5 μm) was used as the stationary phase (Macherey-Nagel, GmbH & Co. KG, Düren, Germany). A system of two solvents was used as the mobile phase, i.e., 1% aqueous solution of formic acid (solvent A) and methanol (solvent B) in a gradient mode of operation: 0–10 min 10% solvent B at a flow rate of 1 mL/min, 10–20 min 25% solvent B at a flow rate of 0.8 mL/min, 20–30 min 45% solvent B at a flow rate of 0.7 mL/min, 30–35 min 45% solvent B at a flow rate of 1 mL/min, 35–40 min 70% solvent B at a flow rate of 1 mL/min, 40–46 min 100% solvent B at a flow rate of 1 mL/min, and 46 min-to-end 10% solvent B at a flow rate of 1 mL/min. The amount of sample used was 10 μL, and all chromatograms were recorded at three wavelengths: 280 nm, 330 nm and 350 nm. Standard solutions of the following substances were used to identify and quantify the components of the extract: caffeic acid, gallic acid, chlorogenic acid, *trans*-cinnamic acid, rosmarinic acid, *p*-coumaric acid, ferulic acid, quercetin, quercitrin, and rutin. All listed substances were obtained from Sigma Aldrich (Sigma Aldrich, Burlington, MA, USA).

Based on the literature data, the presence of coumarin was highly expected; so, another chromatographic procedure was used in order to identify and quantify this compound. The procedure used was previously described in Celeghini et al. [10]. Briefly, a reversed-phase column (Nucleosil C18, size 250 mm × 4.0 mm and particle size of 5 μm) (Macherey-Nagel, GmbH & Co. KG, Düren, Germany) was used as the stationary phase, while the mobile phase was a system of two solvents in the isocratic work mode, namely a mixture of acetonitrile:water (40:60, *v*/*v*). The flow of the mobile phase was set to 1 mL/min, the total amount of sample used was 20 μL, and the chromatogram was recorded at a wavelength of 274 nm. For the identification and quantification of coumarin in the examined extract, the external standard method was used, and the coumarin standard was purchased from the company Sigma Aldrich (Sigma Aldrich, Burlington, MA, USA).

Solutions for the mobile phase were prepared fresh on each individual day when the analysis was performed and filtered through a nylon filter with a pore size of 0.45 μm. The appropriate software Agilent OpenLAB Chemstation v.A.01.05 (Agilent Technologies, Santa Clara, CA, USA) was used for data processing, and the obtained results are expressed as milligrams per gram of dry extract.

### 2.3. Formulation of Semi-Solid Products: Gel and Cream

The preparation of gel started with soaking carbomer 940 in water for 24 h, followed by stirring using the IKA RW 20 digital propeller laboratory mixer (IKA^®^-Werke GmbH & Co. KG, Staufen, Germany). Propylene glycol and triethanolamine solution were added successively to this Carbopol dispersion, and the formed gel was mixed homogenously while avoiding air entrapment. The extract was added in the amount of 2 g to the prepared gel base to achieve a concentration of 2% [11].

The cream base was made by separately mixing oil- and water-phase components heated up to 80 ± 0.5 °C. The oil phase included stearic acid, cetyl alcohol, cetearyl alcohol, and sweet almond oil, while the water phase included water, glycerol, polysorbate 60, and tri-ethanolamine. Once both phases reached the same temperature, the oil phase was slowly added to the water phase under moderate agitation until the mixture’s temperature dropped to 40 °C. After that, the mixture was cooled to room temperature, forming a semi-solid cream base. In the cooling-down stage, the MOE cream was preserved with phenoxyethanol at the concentration of 0.8% and an extract of *M. officinalis* was added in an amount of 2 g to make a concentration of MOE of 2% (Table 1) [12].

### 2.4. Assessment of Physical Properties of Gel and Cream Containing M. officinalis Extract

#### 2.4.1. Determination of Organoleptic Properties

The organoleptic properties included the determination of color, odor, and consistency 24 h after cream and gel preparation. Odor was determined by spreading the formulation samples on a thin glass plate, while color was recorded by spreading a sample on a thin layer on a white paper to maximize the color contrast and comparing the color with the gel and cream bases [13]. 

#### 2.4.2. Determination of the pH Values

The pH values of the examined formulations were determined using a digital pH meter (Mettler Toledo, Columbus, OH, USA), calibrated using a standard buffer solution at 22 ± 2 °C. All measurements were replicated three times [14].

#### 2.4.3. Determination of the Electrical Conductivity

The electrical conductivity (σ) of the examined formulations was determined using a conductivity meter (Eutech CON 700, Thermo Fisher Scientific, Shanghai, China) at 22 ± 2 °C. All measurements were replicated three times [15].

#### 2.4.4. Assessment of Long-Term Stability of the *M. officinalis* Extract-Based Formulations

The long-term stability of the semi-solid formulations with *M. officinalis* extract was assessed by the determination of the organoleptic characteristics and alterations in pH and electrical conductivity values during the storage period. The samples were stored at room temperature, i.e., 22 ± 2 °C, for 6 months. The sampling was conducted after 7, 90, and 180 days of cream and gel storage [16].

#### 2.4.5. Centrifugation Test

The cream and gel samples were centrifuged twice at 3000 rpm for 15 min by using a laboratory centrifuge (Hettich Mikro 120, Germany). Centrifugation was performed at room temperature 22 ± 2 °C for 24 h after the preparation of the semi-solid formulations. Each sample was subjected to visual inspection in order to detect any changes, such as phase separation [17].

#### 2.4.6. Rheological Characterization of Semi-Solid Formulations

The rheological characterization of semi-solid formulations was determined by Anton Paar Rheometer (MCR 102 e) at a constant temperature of 25 ± 0.1 °C, using a serrated plate–plate P35/Ti/SE measuring geometry (the gap thickness between the plates was 0.5 mm) [18].

### 2.5. In Vivo Experiments

#### 2.5.1. Ethical Statement

This investigation was conducted at the Center for Preclinical and Functional Investigations of the Faculty of Medical Sciences, University of Kragujevac, Serbia. The study protocol was performed in accordance with the regulations of the Faculty’s Ethical committee for the welfare of laboratory animals and principles of the Good laboratory practice and European Council Directive (86/609/EEC).

#### 2.5.2. Animals

Healthy male *Wistar albino* rats (eight weeks old, body weight of 200–250 g) were included for the assessment of the skin tolerance and anti-inflammatory activity of *M. officinalis*-based semi-solid formulations. The rats were kept under controlled conditions (temperature of 22 ± 2 °C, cycle of light: darkness 12:12 h), while water and food were available ad libitum. 

#### 2.5.3. Acute Dermal Irritation of Semi-Solid Formulations with *M. officinalis* Extract

The test was conducted according to the Organization for Economic Cooperation and Development (OECD) guidelines 404 [19].

Firstly, the fur was removed by closely clipping the dorsal area, and after one day, the test formulations were applied to a small area of the skin. The animals (*n* = 12) were divided into the following groups:

MOEC—animals treated with 2% *M. officinalis* extract-based cream;

MOEG—animals treated with 2% *M. officinalis* extract-based gel;

BC—animals treated with the cream base;

BG—animals treated with the gel base.

The tested formulations were applied topically in an amount of 500 mg to the shaved area. After that, the rats were placed in individual cages. The animals were observed with special attention during the first 4 h after the administration of the preparation, after which they were observed once a day for a period of 14 days. The rats were observed and we recorded if mortality or any signs of toxicity, such as tremors, salivation, convulsions, or diarrhea, occurred. The occurrence of adverse effects on the skin, such as edema and erythema, was also monitored based on the scoring system [11,19].

#### 2.5.4. Anti-Inflammatory Effects of Semi-Solid Formulations with *M. officinalis* Extract in an Animal Model

A carrageenan-induced rat paw edema model was used for the assessment of the anti-inflammatory potential of the cream and gel based on *M. officinalis* extract. Inflammation in all rats was induced by the intraplantar injection of 1 mL of 0.5% carrageenan saline in the middle part of left hind paw [20]. 

Before the beginning of experiment, *Wistar albino* rats (*n* = 56, 8 per group) were randomly divided into seven groups:

CTRL group—rats with no treatment (negative control);

BC group—rats were treated with the cream base;

BG group—rats were treated with the gel base;

MOE group—rats were treated with 2% solution of *M. officinalis* extract dissolved in water;

MOEC group—rats were treated with 2% *M. officinalis* extract-based cream;

MOEG group—rats were treated with 2% *M. officinalis* extract-based gel;

HC—positive control involved rats treated with hydrocortisone ointment 1%.

All the tested formulations, cream and gel bases, cream and gel based on *M. officinalis*, as well as the hydrocortisone ointment were administered in an amount of 0.3 g 60 min before inducing inflammation and gently rubbed 50 times with the index finger. An hour after the application of the aforementioned formulations, rats from all groups received a subplantar injection of carrageenan (0.5%, 1 mL in the plantar surface of the left hind paw) for inflammation induction. 

In order to quantify the anti-inflammatory potential of the formulations, the thickness of the left paw tissue of each rat was measured at the following time intervals: immediately before inducing inflammation (moment 0) and 1, 2, 3, and 4 h post-carrageenan injection (moments 1, 2, 3, and 4, respectively). The tissue thickness was measured in the middle of rat paw using a digital vernier caliper (Aerospace, China). The percentage of the inhibition of paw edema was calculated according to the formula:

% Inhibition = 100 × [1 − (Yt/Yc)]

where Yt = average increase in paw thickness in the treated group of rats between two measurement moments and Yc = average increase in paw thickness in the untreated group of rats (CTRL) between two measurement moments [20].

### 2.6. Statistical Analyses

The statistical analysis of the obtained data was performed by IBM SPSS 20.0 for Windows. The Kolmogorov–Smirnov and Shapiro–Wilk tests were used to examine the normality of data distribution. Data are expressed as the means ± standard deviation (X ± SD), and the differences between groups were analyzed by a one-way analysis of variance (ANOVA), followed by the Bonferroni test. The differences were considered statistically significant when the *p*-value was lower than 0.05. 

### 2.7. In Silico Studies of Anti-Inflammatory Effects of the Most Abundant Compounds from M. officinalis Extract

#### 2.7.1. Molecular Docking Study

The binding affinity of coumarin, *p*-coumaric acid, gallic acid, and quercetin against COX-1 and COX-2 enzymes was determined using the molecular docking study in the AutoDock 4.2 software [21]. The crystal structures of COX-1 (PDB ID: 3N8Y [22]) and COX-2 (PDB ID: 1PXX [23]) were downloaded from the RCSB Protein Data Bank. BIOVIA Discovery Studio [24] was utilized to prepare target receptors for molecular docking by removing co-crystallized ligands, water molecules, and cofactors from the native crystal structure. Kollman partial charges along with polar hydrogens were added in the graphical user interface AutoDockTools [21]. Docking calculations were performed on the chain A of COX-1 and COX-2, using the Lamarckian Genetic Algorithm (Lamarckian GA) for the rigid protein–flexible ligand docking protocol. The parameters of the Lamarckian GA were specified using the default settings. The binding sites of COX-1 and COX-2 were determined based on co-crystallized ligand (diclofenac (DIC)) coordinates. The search area with a grid point spacing of 0.375 Å was set as a grid box with the dimensions of 40 × 44 × 36 Å^3^ in the -x, -y, and -z directions, respectively, for COX-1 and COX-2 enzymes to encompass the binding site and allow the ligand to change its conformation freely. The three-dimensional interactions between the enzymes and the best docked poses of the tested compounds were analyzed and visualized using Discovery Studio 21.1.0.20298 [24] and Pymol 2.4.1 [25]. The AutoDock 4.2 calculates the free binding energy according to the following equation.

ΔG_bind_ = ΔG_vdw_ + _Hbond_ + _desolv_ + ΔG_elec_ + ΔG_total_ + ΔG_tor_ − ΔG_unb_

where ΔG_bind_ is the estimated free binding energy and ΔG_vdw_ + _Hbond_ + _desolv_ represents the sum of the energies of dispersion and repulsion (ΔG_vdw_), hydrogen bond (ΔG_Hbond_), and desolvation (ΔG_desolv_). ΔG_elec_ is the electrostatic energy, ΔG_total_ is the final total internal energy, ΔG_tor_ is the torsional free energy, and ΔG_unb_ is the unbound system’s energy.

Ligand efficiency (LE) expresses the binding energy per heavy atom of ligand to the target protein.

LE = ∆G_bind_/N

where N is the number of the ligand’s heavy atoms.

#### 2.7.2. Molecular Dynamics Study

Molecular dynamics simulations were performed for the complexes DIC-COX-1, DIC-COX-2, QUE-COX-1, and QUE-COX-2 using the Schrödinger Desmond 2020-4 software [26]. The selected complexes were refined and optimized by the OPLS3e force field [27]. The solvation of the tested systems was carried out using the TIP3P water model [28], while their neutralization was performed with 0.15 M NaCl. MD simulations lasting 20 ns that were set up at constant pressure (1.01325 bar) with recording intervals of 4.8 ps for trajectory and 1.2 ps for energy. The equilibration of the system under the NPT ensemble was conducted at 300 K using OPLS3e force field. The Prime Molecular Mechanics/Generalized Born Surface Area (MM/GBSA) method [29] was utilized for the estimation of the average free binding energy (ΔG_avg_) for frames from the 10 ns of system trajectories. The prime module of Schrödinger tool calculates the ΔG_avg_ value according to the following equation:

MM/GBSA ΔG_avg_ = G_complex_ − G_protein_ − G_ligand_

## 3. Results and Discussion

### 3.1. Chemical Composition of M. officinalis Extract

**Figure 1:**
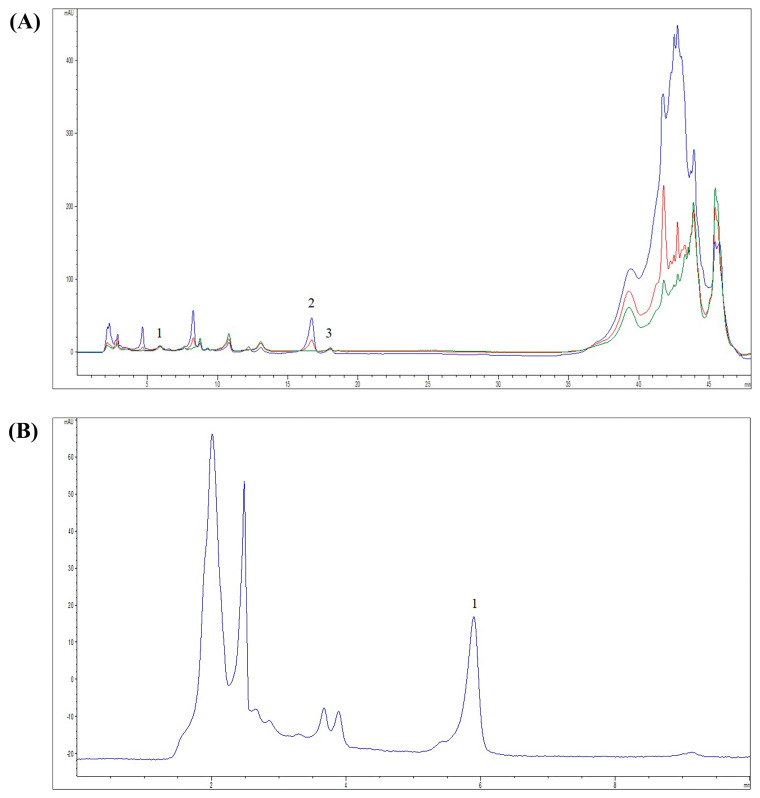
HPLC-DAD chromatograms of *M. officinalis* extract. (**A**) HPLC-DAD chromatograms with detection at 280 nm (blue line), 330 nm (red line), and 350 nm (green line). Identified compounds: 1—GALA, 2—COUMA and 3—QUE; (**B**) HPLC-DAD chromatogram with detection at 274 nm; Identified compound: 1—COUM.

The extraction yield was 11.58% and the obtained extract had a dark brown color. The chemical composition of the *M. officinalis* ethanolic extract is shown in Table 1. The findings suggest the presence of various bioactive molecules, while the most abundant compounds are coumarin (COUM), *p*-coumaric acid (COUMA), gallic acid (GALA), and quercetin (QUE) (Figure 1, Table 2). 

### 3.2. Physicochemical Characterization of M. officinalis Extract-Based Formulations

#### 3.2.1. Organoleptic Characteristics and Physical Appearance of the Formulations

**Figure 2:**
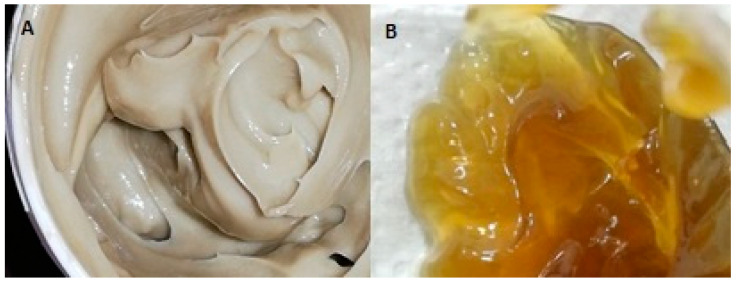
Photographs of the *M. officinalis* extract-based formulations: (**A**) MOEC; (**B**) MOEG.

The cream base was white and homogenous; however, after the addition of the *M. officinalis* extract, it turned into a brown color. While the cream base had no odor, after incorporation of the extract, the cream had a pleasant odor that was characteristic for the extract, i.e., refers to the distinctive smell that is naturally present in the *M. officinalis* extract. It has been considered that the coumarin content in *M. officinalis* extract contributes to the pleasant, sweet, hay-like scent with undertones of vanilla [30]. Similarly, the gel base was transparent with no odor, while the addition of the extract turned it into a light brown color with the odor characteristic for this plant extract. Additionally, both formulations had a semi-solid consistency (Figure 2). 

Regarding the stability of the formulations, no change in the color, odor, and consistency of cream and gel occurred during the follow-up period of 6 months, as shown in Table 2. Moreover, neither the cream nor the gel showed any phase separation during the studied time period (Table 3). Furthermore, stearic acid, which is used in the formulation of creams, can contribute to the stability of the product in the long term. On the other hand, carbomer-based products, when formulated properly and stored under suitable conditions, tend to demonstrate a good long-term stability [31].

#### 3.2.2. pH Values and Electrical Conductivity of the Formulations

The pH values of cream and gel with *M. officinalis* extract are presented in Table 4. There was a significant decrease in the pH value in both the cream and gel after 6 months of storage, while there was no significant alterations after 2 months. Importantly, the pH of the cream formulation ranged from 5.15 to 5.98, as expected due to the stearic type of cream, while the gel formulation ranged from 7.15 to 6.88. This pH range shows that both the cream and gel formulations are suitable for skin application and would not produce either irritation of the skin or impairment in the skin natural barrier. 

Another important parameter for the assessment of formulation stability is measuring the electrical conductivity since alterations in its values over time provide insights into physical and chemical properties and predict shelf-life and performance during storage conditions. Changes in the gel conductivity reflect ion transport properties, swelling behavior, and structural integrity, while, in cream, they may suggest phase separation or the instability of the emulsion [15,32]. Our findings suggest that the electrical conductivity did not change significantly over time (*p* > 0.05) and was in the range of 170.7–177.6 µS for the gel with *M. officinalis* extract and 41.2–39.5 µS for the cream with *M. officinalis* extract. The gel with *M. officinalis* extract had higher values of electrical conductivity, probably due to the structure and higher amount of water compared to the cream with *M. officinalis* extract.

#### 3.2.3. Centrifugation Test

The centrifugation of pharmaceutical formulations, such as creams and gels, has been proposed as a valuable test for the discovery of the preparation instability, including decomposition, phase separation, creaming, and sedimentation. In fact, during this test, samples of the gel and cream were subjected to stress conditions that mimic the elevated gravity force and mobility of particles, which provides insights into whether skin products remain stable or require reformulation [33,34]. In our study, after the centrifugation of both the cream and gel based on *M. officinalis* extract, there was no noticeable decomposition, separation, or precipitation of the components of the formulations. 

#### 3.2.4. Rheological Characterization

**Figure 3:**
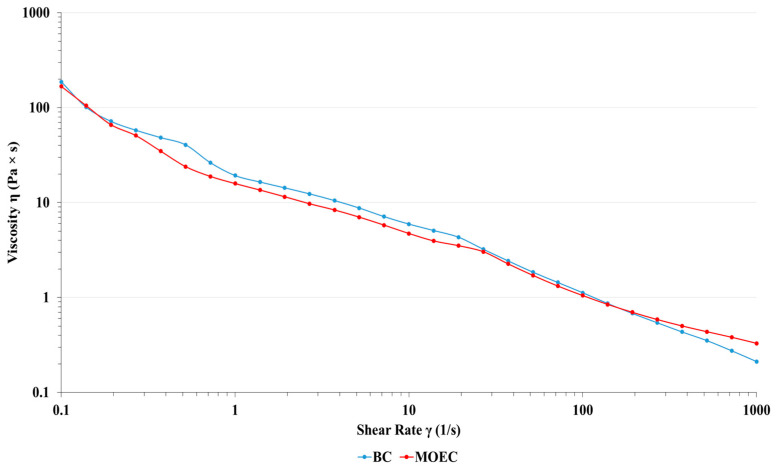
Viscosity as a function of the shear rate for the cream. The curves describe the flow behavior of the sheared and time-independent fluid. BC—cream base; MOEC—*M. officinalis* extract-based cream.

**Figure 4:**
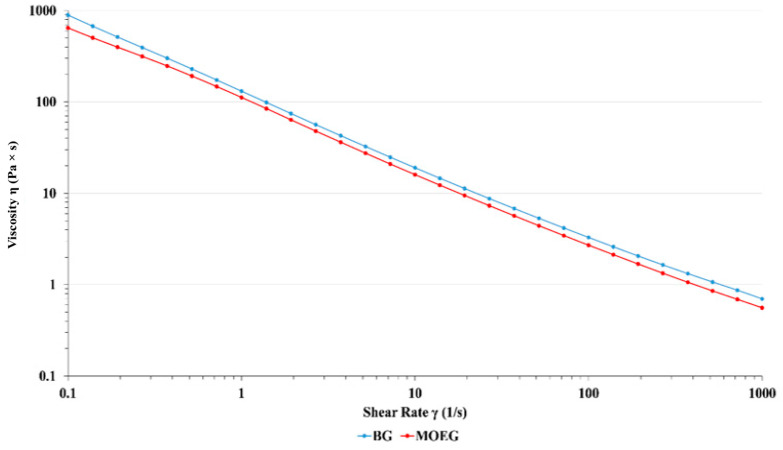
Viscosity as a function of the shear rate for the gel. The curves describe the flow behavior of the sheared and time-independent fluid. BG—gel base; MOEG—*M. officinalis* extract-based gel.

Figure 3 and Figure 4 represent the flow curves that describe the dependence of the viscosity of the cream and gel with *M. officinalis* on the shear rates at the constant temperature of 25 ± 0.1 °C. Proper viscosity is of great importance since it enables the retention of the cream and gel at the site of application for a sufficient time [35]. Both formulations exhibited a non-Newtonian behavior characterized by viscosity dependence under the applied shear conditions. 

The results shown in Figure 3 and Figure 4 demonstrate that the developed semi-solid formulations with *M. officinalis* extract show a shear thinning behavior with the viscosity decreasing as the shear rate increased. This pseudoplastic rheological behavior is suitable for products intended for dermal application, since it enables the desired application’s spreadability and maintenance on the skin [36]. The addition of *M. officinalis* extract into the cream and gel did not affect the rheological behavior. 

### 3.3. Acute Dermal Toxicity of the M. officinalis-Based Formulations

Our study revealed that neither the gel nor cream exert any signs of acute dermal toxicity, such as erythema, edema, and other clinical toxicity manifestations, when applied in a concentration of 2%. 

### 3.4. Anti-Inflammatory Activity of M. officinalis-Based Formulations in a Rat Model

**Figure 5:**
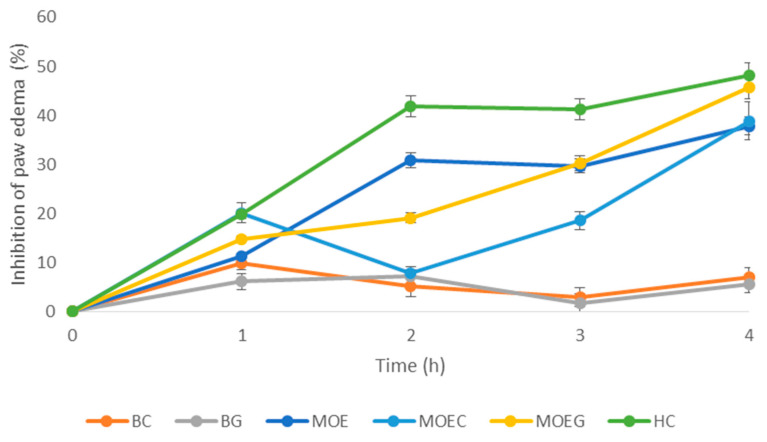
Percentage of paw edema inhibition in rats treated with the formulations. MOE—*M. officinalis* extract; BC—cream base; BG—gel base; MOEG—*M. officinalis* extract-based gel; MOEC—*M. officinalis* extract-based cream; HC—hydrocortisone.

The results of the in vivo anti-inflammatory activity of the *M. officinalis*-based formulations are shown in Table 5 and Figure 5.

After the acute dermal irritation study confirmed no dermal toxicity of the formulated semi-solid products, we continued the assessment of the anti-inflammatory potential of the *M. officinalis*-based formulations in a carrageenan-induced rat model of inflammation. A large body of evidence suggests this model as a reproducible and widely utilized for the investigation of the anti-inflammatory activity of novel formulations [20]. Our findings clearly show that the carrageenan injection led to a significant rise in the paw edema compared to the basal values (point 0). On the other hand, paw edema, which represents an indicator of inflammation, was markedly reduced after the application of both the cream and gel with *M. officinalis*. On the contrary, the rats who received only the gel base and cream base did not experience any change in the inflammation process, as in the control untreated rats. The strong rat paw edema reduction was noticed in the rats treated with MOEG and MOEC, which was manifested by a significant inflammation inhibition of 45.62% and 38.84%, respectively, at the 4th hour following inflammation induction. Importantly, the anti-inflammatory effect of MOEG was comparable to the effect achieved by the hydrocortisone ointment 1% (45.62% vs. 48.21% percentage inhibition, respectively).

Namely, immediately after the injection of carrageenan, the rats experienced edema, erythema, and hyperalgesia due to a release of histamine, serotonin, tachykinin, bradykinin, and other pro-inflammatory agents [37]. According to the literature data, prostaglandin production occurs later, after 2–3 h, and it has been proposed that the carrageenan injection is responsible for TNF-α and IL-1 β generation, which stimulate prostaglandin synthesis by COX-2 [37,38]. In our research, the maximum of paw edema in all groups was reached at 3 h, which is in accordance with previous studies. Additionally, the strongest decrease in rat paw edema was achieved in the 3rd and 4th hours in rats treated with *M. officinalis*, thus suggesting that the applied topical formulations exerted the peak of their anti-edematous effect during the second phase of inflammation, at the time when the edema is maximally developed [38,39,40]. Therefore, we might assume that one of the reason behind the anti-inflammatory property lies in their potential to modulate the immune response partially mediated through the inhibition of COX-2. The achieved benefits of MOEG and MOEC during the delayed phase of inflammation (the 3rd and 4th hours) is a consequence of the interference of the compounds from the extract with the biosynthesis of prostaglandins. The superior effects of the gel with *M. officinalis* extract in our research might be explained by the presence of carbomer that enables the preparation of a low irritancy formulation with optimal aesthetics and active compound penetration [41]. The similar efficacy of MOEG compared to the corticosteroid ointment in the reduction in redness, swelling, and itching associated with inflammation highlights this novel formulation as a promising alternative for the treatment of skin conditions such as eczema, psoriasis, and dermatitis. On the other hand, due to its natural contents, MOEG can be used as a safer treatment for inflammation, especially for long-term application without concerns about dependency or rebound effects, which can occur with corticosteroids [42,43]. However, future studies are necessary in order to provide complete insights into the mechanisms of novel preparations based on *M. officinalis* extract in the attenuation of inflammation response.

### 3.5. In Silico Assessment of Anti-Inflammatory Activity of the Major Compounds from M. officinalis Extract

#### 3.5.1. Molecular Docking Analysis

**Figure 6:**
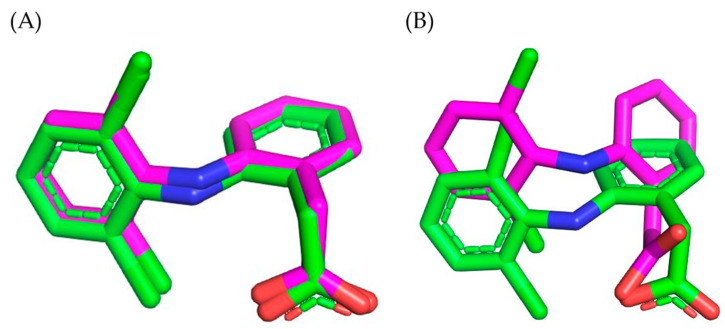
Overlapping of the native (green) and re-docked binding poses (magenta) of DIC in the active sites of: (**A**) COX-1; (**B**) COX-2.

Given the fact that the analyzed extract of *M. officinalis* demonstrated significant anti-inflammatory activity, the binding affinities of the four dominant compounds in the extract, COUM, COUMA, GALA, and QUE, for COX-1 and COX-2 were assessed using a molecular docking study. To establish the validity of the docking protocol, the co-crystallized ligand, DIC, was removed from COX-1 and COX-2 and then remodeled into the active sites of the target enzymes. The calculated RMSD (root-mean-square deviation) values (0.32 Å for COX-1 and 1.77 Å for COX-2) confirmed the validity of the conducted docking protocol (Figure 6A,B).

The binding parameters for the best docked conformations of the investigated compounds are presented in Table 6. The lower value of the estimated free binding energy (ΔG_bind_) and the inhibition constant (K_i_) indicate a stronger interaction of the tested compound and enzyme and, therefore, a stronger inhibition. The binding energy ranges of the tested compounds were between −17.32 and −28.37 kJ/mol and between −15.98 and −29.62 kJ/mol for COX-1 and COX-2, respectively. The calculated inhibition constants were consistent with these findings, with values ranging from 10.67 to 930.56 µM for COX-1 and from 6.43 to 1570.00 µM for COX-2. Notably, QUE exhibited the lowest values of inhibition constant in the micromolar range during molecular docking into the active sites of COX-1 and COX-2, which were lower but still comparable with the values shown by DIC. On the other hand, according to the free binding energies, COUM, COUMA, and GALA bind to COX-1 and COX-2 with a lower affinity compared to DIC.

**Figure 7:**
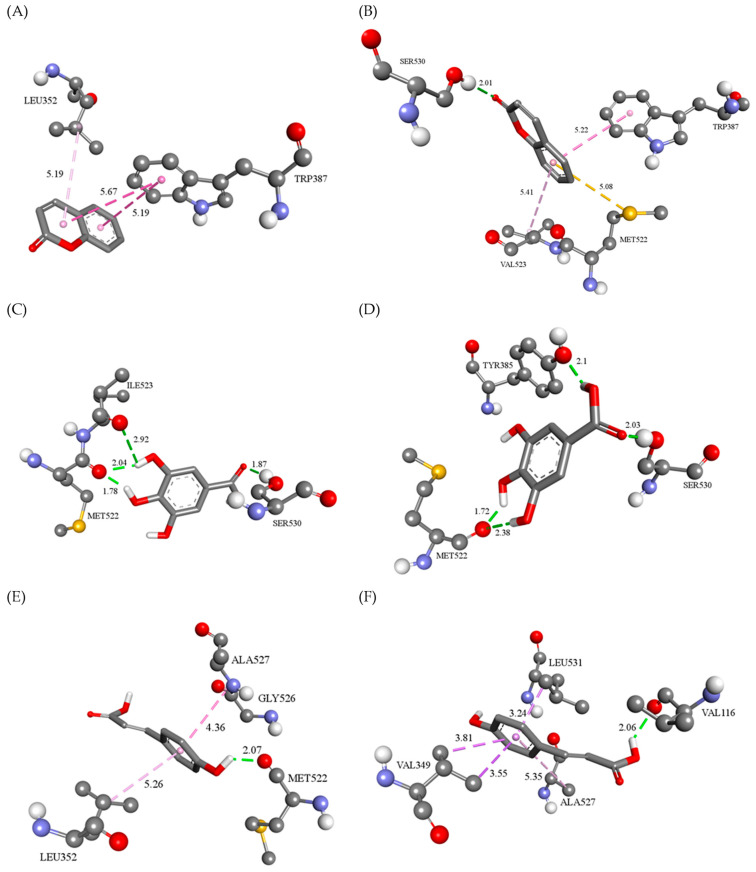
Three-dimensional display of: (**A**) COUM binding interactions in the active sites of (**B**) COX-1 and COX-2; (**C**) GALA binding interactions in the active sites of (**D**) COX-1 and COX-2; and (**E**) COUMA binding interactions in the active sites of (**F**) COX-1 and COX-2. The conventional hydrogen bonds (green dotted lines), π-sulfur (orange dotted lines) interactions, and hydrophobic interactions (pink dotted lines) are presented, as well as their bond lengths.

**Figure 8:**
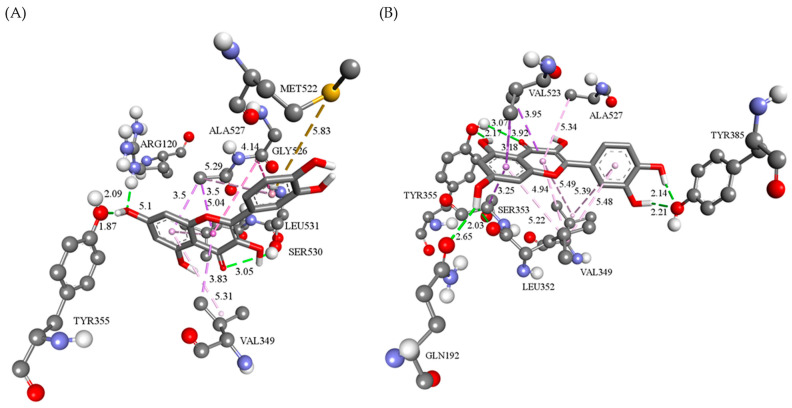
Three-dimensional display of: (**A**) QUE binding interactions in the active sites of (**B**) COX-1 and COX-2. The conventional hydrogen bonds (green dotted lines), π-sulfur (orange dotted lines) interactions, and hydrophobic interactions (pink dotted lines) are presented, as well as their bond lengths.

The molecular fitting of COUM into the active sites of COX-1 and COX-2 revealed the following types of non-covalent interactions: hydrogen bonds (SER530), π-sulfur (MET522), and hydrophobic (LEU352, TRP387, and VAL523) contacts (Figure 7A,B). GALA binds to COX enzymes, thereby predominantly achieving hydrogen bonds with the residues of TYR, MET, ILE, and SER in positions 385, 522, 523, and 530, respectively. Two ortho-hydroxyl groups of GALA act as hydrogen bond donors (bond lengths range from 1.78 to 2.92 Å), while carboxylic group is a hydrogen bond acceptor in interactions with SER530 (bond lengths range from 1.72 to 2.10 Å) (Figure 8A,B). In contrast to the previously mentioned compound, COUMA predominantly achieves a π-alkyl type of hydrophobic interactions with residues LEU352, GLY526, and ALA527 in the active site of COX-1 and with the residues VAL349, ALA527, and LEU531 in the active site of COX-2. In addition, para-hydroxyl and carboxyl groups of *p*-coumaric acid form two hydrogen bonds with carbonyl oxygen atoms from the peptide bonds of residues MET522 in COX-1 (bond length of 2.07 Å) and VAL116 in COX-2 (bond length of 2.06 Å) (Figure 7A–F).

On the other hand, according to the free binding energy values, QUE exhibited a comparable binding affinity towards COX enzymes (ΔG_bind_ = −28.37 kJ/mol for COX-1 and ΔG_bind_ = −29.62 kJ/mol for COX-2) in comparison to DIC (ΔG_bind_ = −33.01 kJ/mol to COX-1 and ΔG_bind_ = −31.04 kJ/mol to COX-2). Specifically, in the active site of COX-1, the C=O group of the QUE 4*H*-hromen core forms an identical hydrogen bond with residue SER530 as the carbonyl oxygen atom of DIC [19]. Furthermore, QUE achieves two more non-covalent polar contacts with residues ARG120 and TYR355. The high affinity of QUE for COX-1 is also contributed to by the grid of hydrophobic interactions formed by the 4*H*-hromen core and phenyl ring. The residues involved in the formation of weak hydrophobic interactions are VAL349, ALA527, LEU531, and GLY526 that form π-σ, π-alkyl, and amide-π stacked interactions. Finally, the residue MET522 forms a π-sulfur interaction with the dihydroxyphenyl moiety of QUE (Figure 8A).

During the molecular modeling of QUE into the COX-2 active site, hydrogen bonds are formed with four amino acid residues. Namely, the hydroxyl group in position 5 of the QUE benzopyran system acts as a hydrogen bond donor when interacting with the GLN192 and LEU352 residues. Tyrosine in position 355 simultaneously forms two hydrogen bonds, one with the hydroxyl group in position 5 and the other with the carbonyl oxygen of benzopyran-4-one. Moreover, the two hydroxyl groups of the QUE benzene ring are proton donors during the formation of two hydrogen interactions with the TYR385 residue. The remaining interactions are hydrophobic, and QUE forms them with the amino acid residues VAL349, SER353, VAL523, and ALA527. Interactions with VAL349 and ALA527 belong to the π-alkyl type, while other residues establish π-sigma types of hydrophobic interactions (Figure 8B).

**Figure 9:**
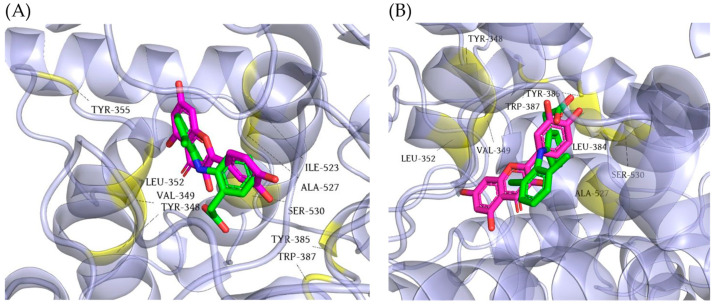
The mutual binding orientation of DIC (colored green) and the best docking pose of QUE (colored magenta) in the active sites (colored yellow) of: (**A**) COX-1; (**B**) COX-2.

The mutual binding orientation of DIC and the best modeled docking poses of QUE in the active sites of COX-1 and COX-2 are shown in Figure 9.

The values of the LE presented in Table 5 illustrate that this parameter is not the main determinant of the free binding energy value. On the contrary, it is evident that the intermolecular energy substantially contributes to the estimated ΔG_bind_ value due to the formation of a large number of hydrogen and hydrophobic interactions. It is also evident that the higher polarity of QUE and GALA, which is reflected through a larger number of OH groups, significantly affects the larger number of formed hydrogen bonds. The estimated torsional energies were quite similar for DIC and the tested compounds, with the exception of COUM, which has a smaller size and lower flexibility compared to DIC and the other investigated molecules.

In a previously published molecular docking study conducted in the CB-Dock online server (http://cao.labshare.cn/cb-dock/), DIC demonstrated a higher binding affinity towards COX-2 (−33.89 kJ/mol) in comparison to COX-1 (−27.61 kJ/mol), which is opposite to our findings [44]. Vyshnevska and colleagues performed a similar in silico analysis of various herbal compounds’ binding affinities to COX-2 in AutoDock Vina, and COUM, GALA, and QUE showed lower free binding energy values (−30.12, −26.36, and −41.00 kJ/mol, respectively) in relation to our tested compounds [45].

#### 3.5.2. Molecular Dynamics Analysis and MM/GBSA Calculation

MD simulations were carried out to investigate the conformational stability of DIC-COX-1, DIC-COX-2, QUE-COX-1, and QUE-COX-2 complexes, and the best modeled docking pose of DIC was used. 

**Figure 10:**
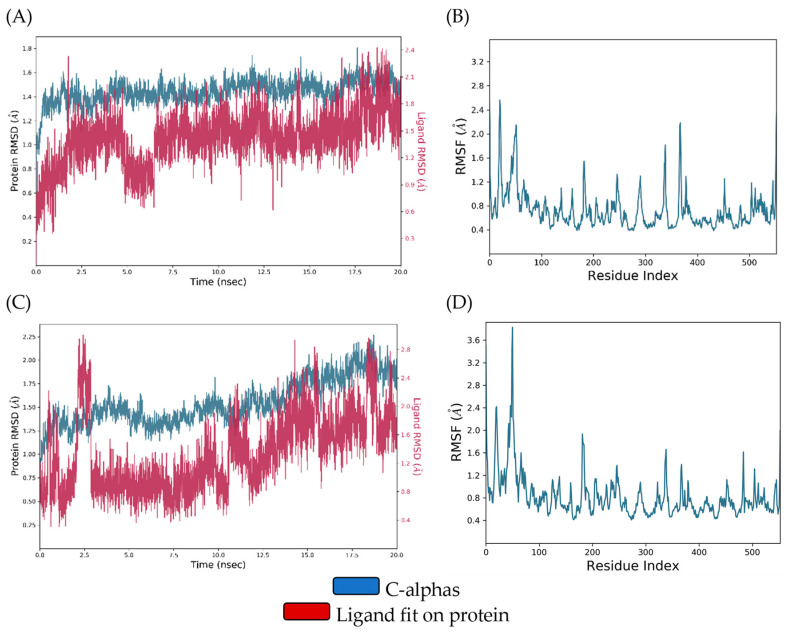
(**A**) RMSD of the Cα of COX-1 and DIC against the simulation time; (**B**) COX-1 RMSF fluctuations of the Cα against the residue index obtained for the DIC-COX-1 complex; (**C**) RMSD of the Cα of COX-1 and QUE against the simulation time; (**D**) COX-1 RMSF fluctuations of the Cα against the residue index obtained for the QUE-COX-1 complex.

The ligand–protein RMSD plot of the DIC-COX-1 complex revealed conformational stability throughout the entire simulation, but DIC showed a certain change in RMSD values in the interval from 4.84 to 6.48 ns. On the other hand, the QUE-COX-1 complex demonstrated RMSD deviations during the first 10 ns and, thereafter, became stable till the end of the simulation. The comparison of DIC-COX-1 and QUE-COX-1 RMSF (root-mean-square fluctuation) diagrams indicates that amino acids involved in ligand binding did not show significant fluctuations. The conformational stability of these residues (ARG120, VAL349, TYR355, MET522, GLY562, ALA527, SER530, and LEU531), discussed in the molecular docking study, is a consequence of the similar binding modes of DIC and QUE (Figure 10A–D).

**Figure 11:**
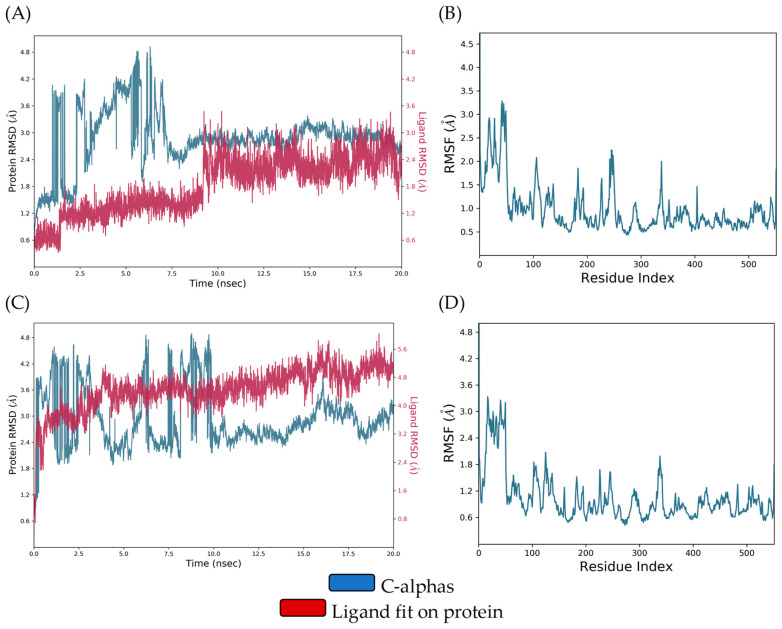
(**A**) RMSD of the Cα of COX-2 and DIC against the simulation time; (**B**) COX-2 RMSF fluctuations of the Cα against the residue index obtained for the DIC-COX-2 complex; (**C**) RMSD of the Cα of COX-2 and quercetin against the simulation time; (**D**) COX-2 RMSF fluctuations of the Cα against the residue index obtained for the QUE-COX-2 complex.

In the first half of the simulation, DIC and COX-2 showed prominent deviations in RMSD values, and then, the complex was stabilized till the end of the simulation. Similarly, the RMSD diagrams of QUE and COX-2 are mutually overlapped, indicating the formation of a relatively stable complex. The RMSF diagram analysis of the DIC-COX-2 and QUE-COX-2 complexes suggests that key binding amino acid residues (GLN192, VAL349, LEU352, SER353, TYR355, TYR385, and ALA527) showed minor local conformational changes due to the analogous binding modes of DIC and QUE (Figure 11A–D).

**Figure 12:**
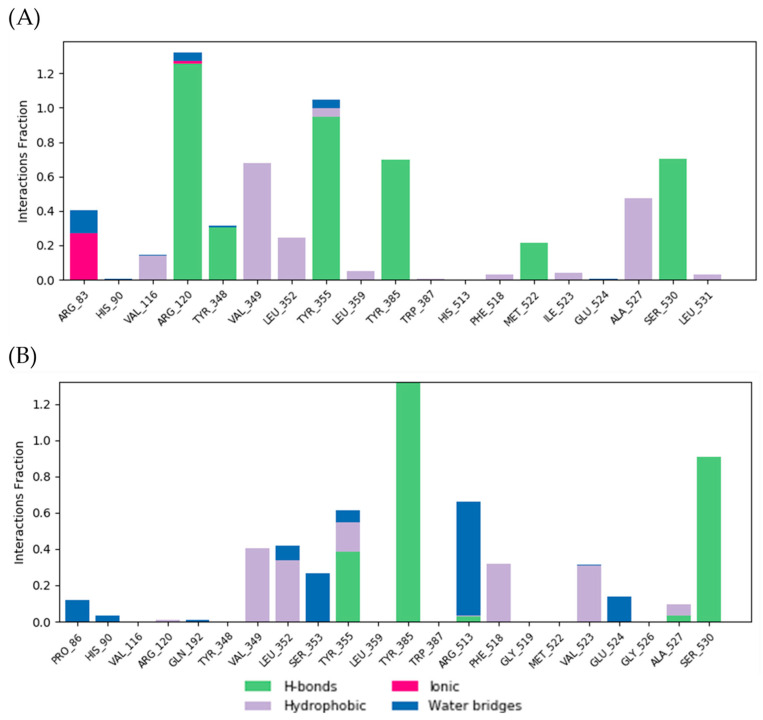
(**A**) QUE-COX-1 and (**B**) QUE-COX-2 contacts during the MD simulation. Green bars (hydrogen bonds), red bars (ionic interactions), gray bars (hydrophobic interactions), and blue bars (water bridges) are presented.

The binding interaction fractions of residues involved in the stabilization of the QUE-COX-1 and QUE-COX-2 complexes during MD simulations are presented in Figure 12. The molecular modeling of QUE into the structure of COX-1 revealed the formation of three major polar interactions with residues ARG120, TYR355, and SER530. These hydrogen bonds predominantly contribute to the stabilization of the QUE-COX-1 complex, whereby the interaction with ARG120 lasted for over 120% of simulation time, the interaction with TYR355 was present near the 100% of the time, while the interaction formed with SER530 was maintained for approximately 70% of the simulation. Due to the formation of multiple hydrophobic interactions, the residues VAL349 and ALA527 stood out as contacts with the highest achieved continuity during 70% and 50% of the simulation, respectively (Figure 12A). As previously stated, the amino acid residue TYR385 of COX-2 formed a double hydrogen bond with QUE; therefore, this type of interaction was present for over 130% of the simulation time. In contrast, the TYR355 residue established a hydrogen polar contact during 38% of the simulation. With regard to hydrophobic contacts, the highest continuity was shown by VAL349, which was present during approximately 40% of the simulation, while LEU352 formed a hydrophobic contact during 34% of the simulation period. The remaining key hydrophobic contacts contributed to the stability of the QUE-COX-2 complex for less than 10% of the time (Figure 12B).

The average free binding energy values (∆G_avg_) of the DIC and QUE complexes with COX enzymes were calculated using the MM/GBSA approach and are presented in Table 7.

The free binding energy values obtained by the molecular docking analysis indicate that COUM, COUMA, and GALA bind to COX-1 and COX-2 with lower binding affinities compared to DIC. In contrast to them, QUE demonstrated a stronger interaction with COX-1 and COX-2, and thereby a comparable inhibitory activity to that of DIC (−33.01 for COX-1 and −31.04 kJ/mol for COX-2). According to the calculated values of the free binding energy, QUE showed a slightly higher binding affinity towards COX-2 than COX-1 (−29.62 vs. −28.37 kJ/mol). The obtained MM/GBSA binding energy for QUE confirmed this result with regard to COX-1, with a significantly higher binding energy calculated for QUE than DIC. However, in contrast to the preliminary molecular docking results, QUE achieved a remarkably low value of MM/GBSA binding energy, suggesting that QUE formed a more stable complex with the COX-2 enzyme in comparison to DIC. The MD simulation results verify the formation of a stable QUE-COX-2 complex and thereby confirm the stability of QUE in the binding pocket of COX-2 during the simulation time.

## 4. Conclusions

The developed gel and cream loaded with the *M. officinalis* ethanolic extract possess suitable physical properties for skin application that were not altered over a 6-month storage period, thus ensuring product stability and consumer satisfaction. None of the semi-solid formulations induce any signs of dermal irritation, while exerting significant potential in inflammation reduction in the carrageenan rat paw edema model. The gel based on the *M. officinalis* extract achieved a superior anti-inflammatory activity that was comparable with the hydrocortisone ointment. The gel application provides a natural and safer alternative to corticosteroid treatment and might be a promising tool for mitigating inflammatory-mediated skin diseases. Based on the calculated free binding energy, average MM/GBSA energy, and MD results, quercetin stands out as a molecule with potent inhibitory affinity against COX-1 and COX-2. In that sense, further in vivo investigations of quercetin activity should be performed to determine its individual anti-inflammatory effect in various biological systems.

## Figures and Tables

**Table 1:** Concentration of all components in the gel and cream formulations.

Formulations	Components	Quantity (g)
MOEG	*M. officinalis* extract	2
	Carbomer 940	0.5
	Propylene glycol	10
	Triethanolamine	q.s.
	Water	ad 100
MOEC	Stearic acid	10
	Cetyl alcohol	2
	Cetearyl alcohol	2
	Polysorbate 60	2
	Sweet almond oil	4
	Glycerol	3
	Phenoxyethanol	0.8
	Triethanolamine	q.s.
	*M. officinalis* extract	2
	Water	ad 100

MOEC—*M. officinalis* extract-based cream; MOEG—*M. officinalis* extract-based gel.

**Table 2:** Retention times and concentration of compounds identified in the *M. officinalis* ethanolic extract.

Compound	Retention Time (t_R_ (min))	Concentration (mg/g DE)	Uncertainty (U)
GALA *	8.27	1.31	0.20
COUMA *	16.67	3.18	0.32
QUE *	18.02	0.36	0.03
COUM ^#^	5.9	10.81	0.86

DE—dry extract; U—expanded measuring uncertainty with coverage factor k = 2; * first chromatography technique used; ^#^—second chromatography technique used; GALA—gallic acid; COUMA—*p*-coumaric acid; QUE—quercetin; COUM—coumarin.

**Table 3:** Organoleptic characteristics and physical appearance of gel and cream with *M. officinalis* extract after 1, 60, and 180 days of preparation and storage at 22 ± 2 °C.

	MOEC			MOEG		
Parameters	7 Day	60 Days	180 Days	1 Day	60 Days	180 Days
Color	Light brown	Light brown	Light brown	Brownish	Brownish	Brownish
Odor	Characteristic odor of the extract	Characteristic odor of the extract	Characteristic odor of the extract	Characteristic odor of the extract	Characteristic odor of the extract	Characteristic odor of the extract
Consistency	Semi-solid	Semi-solid	Semi-solid	Semi-solid	Semi-solid	Semi-solid
Homogeneity	No phase separation	No phase separation	No phase separation	No phase separation	No phase separation	No phase separation

MOEC—*M. officinalis* extract-based cream; MOEG—*M. officinalis* extract-based gel.

**Table 4:** pH values and electrical conductivity of the gel and cream after 1, 60, and 180 days of preparation and storage at 22 ± 2 °C.

		pH	Electrical Conductivity (µS/cm)
MOEC	0 day	5.98 ± 0.09	41.2 ± 0.23
	60 days	5.52 ± 0.11	40.8 ± 0.28
	180 days	5.15 ± 0.15 *	39.5 ± 0.21
MOEG	0 day	7.42 ± 0.10	177.6 ± 1.83
	60 days	7.32 ± 0.09	174.5 ± 1.26
	180 days	6.88 ± 0.14 *	170.7 ± 1.68

Data are shown as mean  ±  SD (*n*  =  3). * statistically significant difference at the *p* < 0.05 level compared to day 0. MOEC—*M. officinalis* extract-based cream; MOEG—*M. officinalis* extract-based cream.

**Table 5:** Anti-inflammatory activity of the *M. officinalis*-based formulations in the carrageenan-induced rat paw edema model.

Rat Paw Thickness (mm) (% of Inhibition)					
Groups	0 h	1 h	2 h	3 h	4 h
BC	1.45 ± 0.31	2.82 ± 0.40 (9.90%)	5.22 ± 0.33 (5.09%)	4.97 ± 0.40 (2.93%)	4.67 ± 0.42 (6.97%)
BG	1.53 ± 0.44	2.94 ± 0.60 (6.07%)	5.11 ± 0.54 (7.09%)	5.03 ± 0.51 (1.75%)	4.74 ± 0.38 (5.57%)
MOE	1.56 ± 0.02	2.78 ± 0.23 (11.80%)	3.80 ± 0.46 (30.90%) *	3.60 ± 0.31 (29.69%) *	3.12 ± 0.23 (37.84%) *
MOEC	1.76 ± 0.17	2.5 ± 0.13 (20.12%)	5.07 ± 0.29 (7.81%)	4.17 ± 0.35 (18.55%) *	3.07 ± 0.21 (38.84%) *
MOEG	1.72 ± 0.06	2.67 ± 0.20 (14.69%)	4.45 ± 0.87 (19.09%)	3.57 ± 0.76 (30.27%) *	2.73 ± 0.16 (45.62%) *
HC	1.64 ± 0.23	2.51 ± 0.35 (19.80%)	3.20 ± 0.18 (41.81%) *	3.01 ± 0.27 (41.21%) *	2.60 ± 0.13 (48.21%) *
CTRL	1.51 ± 0.16	3.13 ± 0.10	5.50 ± 0.17	5.12 ± 0.12	5.02 ± 0.42

Results are presented as the mean value ± standard deviation (*n* = 8). * A statistically significant difference at the level of *p* < 0.05 in relation to the control group. MOE—*M. officinalis* extract; BC—cream base; BG—gel base; MOEG—*M. officinalis* extract-based gel; MOEC—*M. officinalis* extract-based cream; HC—hydrocortisone; CTRL—control.

**Table 6:** Binding parameters for the best docked conformations of the tested compounds with COX-1 and COX-2.

Complex	ΔG_bind_ (kJ/mol)	K_i_ (µM)	ΔG_Intermol_. _Energy_ (_vdw_ + _Hbond_ + _desolv_) (kJ/mol)	ΔG_elec_ (kJ/mol)	ΔG_Final Intermol_. _Energy_ (kJ/mol)	ΔG_total_ (kJ/mol)	ΔG_tor_ (kJ/mol)	ΔG_unb_ (kJ/mol)	LE *
DIC-COX-1	−33.01	1.64	−36.15	−1.46	−37.61	−3.68	4.60	−3.68	−1.90
DIC-COX-2	−31.04	3.66	−35.40	−0.25	−35.60	−3.22	4.60	−3.22	−1.63
COUM-COX-1	−23.68	70.20	−3.51	−0.17	−23.68	0	0	0	−2.15
COUM-COX-2	−23.22	84.60	−22.97	−0.25	−23.22	0	0	0	−2.11
COUMA-COX-1	−19.29	416.74	−23.76	−0.13	−23.89	−0.29	4.60	−0.29	−1.61
COUMA-COX-2	−19.37	404.73	−19.87	−0.98	−23.97	−0.29	4.60	−0.29	−1.61
GALA-COX-1	−17.32	930.56	−21.42	−1.63	−23.05	−6.02	5.73	−6.02	−1.44
GALA-COX-2	−15.98	1570.00	−20.83	−0.92	−21.75	−5.90	5.73	−5.90	−1.33
QUE-COX-1	−28.37	10.67	−34.56	−0.71	−35.27	−9.95	6.9	−9.95	−1.29
QUE-COX-2	−29.62	6.43	−35.73	−0.79	−36.52	−10.00	6.9	−10.00	−1.35

* LE—ligand efficiency; DIC—diclofenac; COUM—coumarin; COUMA—*p*-coumaric acid; GALA—gallic acid; QUE—quercetin.

**Table 7:** MM/GBSA average binding energies (∆G_avg_) of DIC and QUE.

Target	Ligand	MM/GBSA ΔGavg ± SD * (kJ/mol)
COX-1	DIC	−229.4443 ± 18.1586
	QUE	−168.2767 ± 22.0497
COX-2	DIC	−172.9281 ± 10.4182
	QUE	−229.3945 ± 20.5434

* SD—standard deviation.

## Data Availability

The data presented in this study are available in this article.
